# Crooke Cell Adenoma Presenting With Refractory Hypokalemia and Uncontrolled Hypertension

**DOI:** 10.1210/jcemcr/luaf293

**Published:** 2025-12-18

**Authors:** Heng Yeh, Htoo Myat Nge, Karen Racedo Askari, Gerald Feliz Reis

**Affiliations:** Department of Medicine, Bassett Medical Center, Cooperstown, NY 13326, USA; Internal Medicine Residency Program, Memorial Healthcare System, Pembroke Pines, FL 33028, USA; Department of Endocrinology, Memorial Healthcare System, Hollywood, FL 33021, USA; Department of Pathology, Memorial Healthcare System, Hollywood, FL 33021, USA

**Keywords:** Crooke cell adenoma, pituitary microadenoma, refractory hypokalemia, Cushing disease

## Abstract

Crooke cell adenomas (CCAs) are a rare subtype of pituitary corticotroph adenomas with a prevalence of less than 1% of pituitary adenomas. CCAs have variable clinical manifestations and can present as functional adenomas secreting adrenocorticotropic hormone (ACTH), resulting in hypercortisolism, as well as silent invasive macroadenomas. CCAs also exhibit a higher risk for recurrence, less biochemical remission, and normalization of pituitary function after tumor resection compared to other secretory corticotroph adenomas. Refractory hypokalemia and hypertension as manifestations of hypercortisolism are more commonly seen in patients with ectopic ACTH syndrome than pituitary-dependent Cushing syndrome. We present a 39-year-old female who presented with refractory hypokalemia, hypertension, lower extremities edema, and newly diagnosed diabetes mellitus. Evaluation revealed an ACTH-dependent hypercortisolemia from a 5- × 3-mm pituitary microadenoma. She underwent transsphenoidal resection of the microadenoma with resolution of symptoms immediately after surgery and up to 20 months of follow-up. The histopathology of the microadenoma revealed more than 50% of corticotrophs demonstrated Crooke hyaline change compatible with Crooke cell adenoma. To our knowledge, only one case of CCA presenting with a microadenoma and similar clinical manifestations has been reported to date. Our case highlights an atypical and infrequent clinical presentation of CCA.

## Introduction

Crooke cell adenomas (CCAs) are a rare subtype of pituitary corticotroph adenomas and represent less than 1% of pituitary adenomas [1]. CCAs are diagnosed through histopathology when more than 50% of the pituitary adenoma cells exhibit Crooke hyaline change, the massive accumulation of cytokeratin (CK) filaments around the nucleus resulting in a distinctly hyaline appearance in hematoxylin and eosin (H&E) staining. Crooke hyaline change is commonly seen in non-neoplastic corticotrophs in Cushing disease and could be seen in the setting of excessive blood levels of glucocorticoids, such as ectopic adrenocorticotropic hormone (ACTH) syndrome, cortisol-producing adrenocortical tumors, or patients treated with glucocorticoids. The hyaline change of the corticotrophs is assumed to be the response to excessive glucocorticoids and is thought to suppress these cells functionally. The corticotroph adenomas generally do not undergo Crooke hyaline change due to their autonomous hormonal hypersecretion [2].

CCAs exhibit diverse clinical manifestations. In a case series conducted by George et al, 65% of CCAs had features of overt Cushing disease at presentation. In 7 of the 12 cases reported as silent adenomas at presentation, a variety of hypercortisolism features were noticed retrospectively, including truncal obesity and psychiatric disturbances. Visual complaints were noticed in 45% of the cases, and headache was noticed at presentation in 76% of the cases. Most of the CCAs were macroadenomas (81%) and grossly or radiologically invasive (72%) with suprasellar extension (67%) [2]. In another literature review conducted by Giraldi et al, 63.8% of CCAs were functional adenomas, with 83% of these as macroadenomas. All reported biochemically silent CCAs in the same review were macroadenomas [3].

Hypokalemia as a manifestation of Cushing syndrome is more commonly noticed in patients with ectopic ACTH syndrome compared to patients with Cushing disease [4]. We report a case of Crooke cell microadenoma presenting with refractory hypokalemia and resistant hypertension. She underwent transsphenoidal tumor resection after a diagnostic inferior petrosal sinus sampling with complete biochemical and clinical remission up to 20 months of monitoring.

## Case Presentation

A 39-year-old female patient with a medical history of postsurgical hypothyroidism due to a multinodular goiter and polycystic ovary syndrome, presented to the clinic with severe refractory hypokalemia, hypertension, and uncontrolled diabetes mellitus. She had 3 hospitalizations in the past 3 months for hypertensive urgencies, severe hypokalemia with muscle cramping, and lower extremities swelling. She also reported unintentional weight gain of 100 lbs in the past 6 months and increased facial hair growth. Her medications include amlodipine-valsartan 10-320 mg daily, potassium chloride 20 mEq daily, metoprolol succinate 25 mg daily, spironolactone 25 mg daily, levothyroxine 125 mcg daily, metformin 1000 mg twice daily, insulin glargine 60 units daily, and insulin aspart 15 to 20 units before meals. She was only on thyroid hormone replacement therapy 3 months ago. She was using a levonorgestrel intrauterine device for contraception.

Physical examination revealed elevated blood pressure of 170/90 mmHg, body mass index (BMI) of 50.28 kg/m^2^, facial hair around lips and chin, round face, dorsocervical fat pad, central obesity, purple and white stretch marks, acanthosis nigricans on skin folds, and bilateral lower extremities pitting edema up to her knees.

## Diagnostic Assessment

Initial blood tests revealed elevated hemoglobin A1c 9.8% (reference range, < 5.7%), and severe hypokalemia 2.5 mEq/L (SI: 2.5 mmol/L) (reference range, 3.6-5.2 mEq/L [SI: 3.6-5.2 mmol/L]). Hormonal evaluation revealed significantly elevated morning cortisol levels, 24-hour urinary free cortisol, and late-night salivary cortisol levels with elevated ACTH, suggesting an ACTH-dependent hypercortisolemia. Overnight morning cortisol level failed to be suppressed after a dose of 8 mg dexamethasone (Table 1).

**Table 1.** Laboratory tests at time of presentation including hormonal evaluation

Tests	Reference ranges	Results
Hemoglobin A1c	< 5.7%	9.8% (H)
Random plasma glucose	70-140 mg/dL (SI: 3.9-7.8 mmol/L)	238 mg/dL (SI: 13.22 mmol/L) (H)
Potassium	3.6-5.2 mEq/L (SI: 3.6-5.2 mmol/L)	2.5 mEq/L (SI: 2.5 mmol/L) (L)
Sodium	135-145 mEq/L (SI: 135-145 mmol/L)	139 mEq/L (SI: 139 mmol/L)
Blood urea nitrogen (BUN)	6-21 mg/dL (SI: 2.14-7.50 mmol/L)	16 mg/dL (SI: 5.71 mmol/L)
Creatinine	0.59-1.04 mg/dL (SI: 52-92 μmol/L)	0.54 mg/dL (SI: 47.74 μmol/L) (L)
Carbon dioxide (CO_2_)	22-29 mEq/L (SI: 22-29 mmol/L)	30 mEq/L (SI: 30 mmol/L) (H)
Morning cortisol	7-25 μg/dL (SI: 193-690 nmol/L)	60.4 μg/dL and 112 μg/dL (SI: 1666.44 nmol/L and 3090 nmol/L) (H)
Adrenocorticotropic hormone (ACTH)	7.2-63 pg/mL (SI: 1.6-13.9 pmol/L)	264.0 and 309 pg/mL (SI: 58.1 pmol/L and 68.0 pmol/L) (H)
24-hour urinary free cortisol (UFC)	3.5-45 μg/24 hours (SI: 9.7-124 nmol/24 hours)	1107 μg/24 hours (SI: 3055 nmol/24 hours) (H)
Late-night 11 Pm salivary cortisol	< 100 ng/dL (SI: <2.76 nmol/L)	3170 and 4310 ng/dL (SI: 87.48 nmol/L and 118.90 nmol/L) (H)
8 mg overnight dexamethasone suppression test	< 1.8 μg/dL (SI: 49.7 nmol/L) after 8 mg dexamethasone suppression test	62 μg/dL (SI: 1710 nmol/L) (H)
Aldosterone	<21 ng/dL (SI: <583 pmol/L)	<1.0 ng/dL (SI: <27.7 pmol/L)
Renin	0.6-4.3 ng/mL/h (SI: 16.7-119.5 pmol/L/s)	0.460 ng/mL/h (SI: 12.8 pmol/L/s) (L)
Estradiol	Follicular: 30-100 pg/mL (SI: 110-367 pmol/L) Luteal: 70-300 pg/mL (SI: 257-1100 pmol/L)	101.2 pg/mL (SI: 371.4 pmol/L)
Follicle-stimulating hormone (FSH)	Follicular: 2.9-14.6 mIU/mL (SI: 2.9-14.6 IU/L) Midcycle: 4.7-23.2 mIU/mL (SI: 4.7-23.2 IU/L) Luteal: 1.4-8.9 mIU/mL(SI: 1.4-8.9 IU/L)	<0.30 mIU/mL (SI: <0.30 IU/L) (L)
Luteinizing hormone (LH)	Follicular: 1.9-14.6 mIU/mL (SI: 1.9-14.6 IU/L) Midcycle: 12.2-118.0 mIU/mL (SI: 12.2-118.0 IU/L) Luteal: 0.7-12.9 mIU/mL (SI: 0.7-12.9 IU/L)	<0.7 mIU/mL (SI: <0.7 IU/L) (L)
Prolactin	4.8-23.3 ng/mL (SI: 4.8-23.3 μg/L)	9.9 ng/mL (SI: 9.9 μg/L)

The pronounced cortisol hypersecretion with elevated adrenocorticotropic hormone (ACTH) suggested an ACTH-dependent hypercortisolism.

**Figure 1.**
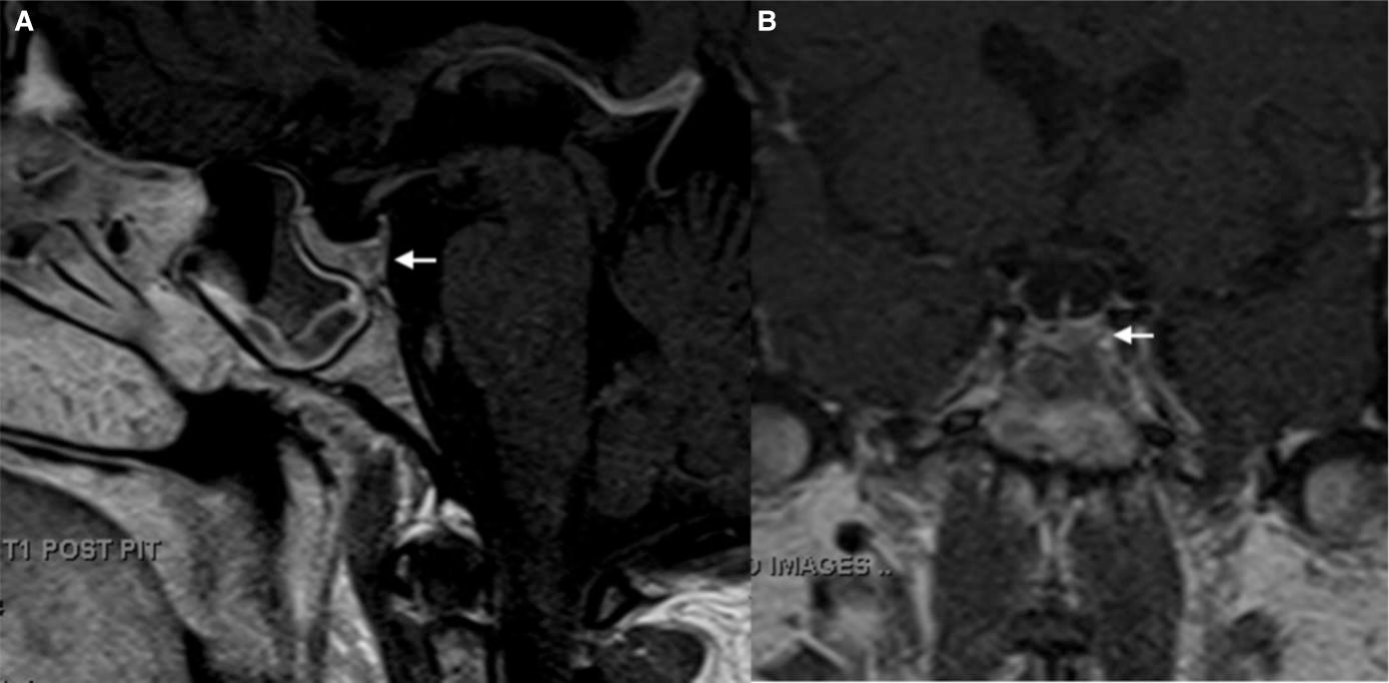
T1-weighted magnetic resonance imaging of the brain (MRI) after intravenous Gadolinium contrast. (A) Sagittal view of the MRI revealed a hypo-enhancing lesion in the pituitary gland, sized 5.3 × 3.3 mm. (B) Coronal view of the MRI revealed a hypo-enhancing lesion on the left side of the sella turcica, causing the asymmetrical appearance. The sphenoid sinus was noticed to be opacified in the MRI.

Magnetic resonance image of the brain (MRI) revealed a hypo-enhancing lesion sized 5 × 3 mm in the pituitary gland, suggesting a pituitary microadenoma (Fig. 1A and 1B). There were no other lesions or tumors noticed in the computed tomography (CT) of the chest, abdomen, and pelvis.

Bilateral inferior petrosal sinus sampling (BIPSS) suggested a central source of ACTH with an elevated central to peripheral ACTH ratio at baseline and after antidiuretic hormone stimulation (Table 2).

**Table 2.** Bilateral inferior petrosal sinus sampling results and interpretation

	ACTH				
Time		Inferior petrosal sampling			
	Peripheral	Right IPS	Left IPS	Central/Peripheral ratio	
				R/P	L/P
Baseline	143 pg/mL (31.5 pmol/L)	1074 pg/mL (236.3 pmol/L)	5 pg/mL (1.1 pmol/L)	7.51	0.035
2 minutes post-ADH	167 pg/mL (36.7 pmol/L)	5433 pg/mL (1195.3 pmol/L)	3937 pg/mL (866.1 pmol/L)	32.533	23.575
5 minutes post-ADH	153 pg/mL (33.7 pmol/L)	4318 pg/mL (950.0 pmol/L)	3921 pg/mL (862.6 pmol/L)	29.222	25.627
10 minutes post-ADH	225 pg/mL (49.5 pmol/L)	2380 pg/mL (523.6 pmol/L)	N/A	10.578	0
15 minutes post-ADH	126 pg/mL (27.7 pmol/L)	1611 pg/mL (354.4 pmol/L)	3289 pg/mL (723.6 pmol/L)	12.786	26.103

Baseline right IPS to peripheral ratio 7.51, more than 2 suggestive of central source of ACTH. Highest right IPS to peripheral ratio 32.5 at 2 minutes post ADH infusion suggestive of central source of ACTH. Left IPS sample was impaired at baseline and 10 minutes post ADH infusion. All samples had prolactin IPS to peripheral ratio > 1.8 indicating correct catheter placement.

Abbreviations: ACTH, adrenocorticotropic hormone; ADH, antidiuretic hormone; IPS, inferior petrosal sampling; L/P, left/peripheral ratio; R/P, right/peripheral ratio.

## Treatment

Her blood pressure remained uncontrolled, requiring frequent hospitalization and emergency department visits. Spironolactone was increased to 100 mg daily, and metoprolol was switched to carvedilol 25 mg twice daily, with additional medications including furosemide 40 mg daily and hydralazine 100 mg 3 times daily. Her potassium supplement was increased to potassium chloride 40 mEq once or twice daily. Insulin requirements continued to increase, requiring over 200 units of insulin daily.

**Figure 2.**
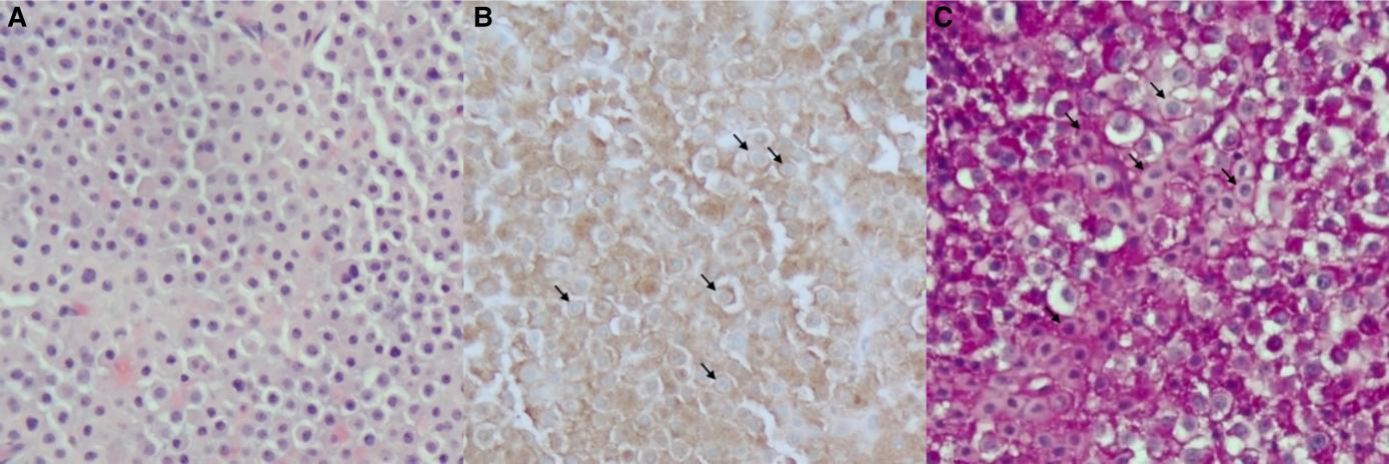
Surgical pathology examination of the resected pituitary adenoma. (A) Hematoxylin and eosin (H&E) stain of the neoplastic cells. (B) Immunohistochemistry for adrenocorticotropic hormone (ACTH) demonstrates diffuse ACTH staining with relocation of the secretory granules to the periphery (arrows). (C) Periodic acid–Schiff (PAS)-positive secretory granules are relocated to the periphery of the cells (arrows) as seen in the ACTH stain, which is supportive of Crooke cell adenoma.

By the time the BIPSS results were available, she was admitted urgently and underwent urgent transsphenoidal endoscopic resection of the pituitary microadenoma. Subcutaneous enoxaparin 40 mg daily was given for deep vein thrombosis prophylaxis during the course of admission. The histopathology of the microadenoma revealed a corticotroph adenoma with positive ACTH immunohistochemical stain, with more than 50% of the corticotrophs demonstrating perinuclear Crooke hyaline change (Fig. 2A-2C).

## Outcome and Follow-Up

She had an immediate reduction in her ACTH and cortisol levels after surgery. The ACTH level decreased from 118 pg/mL (SI: 26 pmol/L) before surgery to 10 pg/mL (SI: 2.20 pmol/L) on postoperative day 1. The serum cortisol level also decreased from 112.6 μg/dL (3107.6 nmol/L) to a nadir of 4.6 μg/dL (127.0 nmol/L) on postoperative day 2. Intravenous (IV) hydrocortisone 50 mg every 8 hours was started on postoperative day 2, and this was later tapered to 20 mg twice daily orally before discharge.

Her blood pressure returned to the 130/80 mmHg range after surgery and only required carvedilol 12.5 mg twice daily for blood pressure control. Potassium chloride was tapered to 20 mEq daily with normalization of potassium level, and she no longer complained of leg cramping. Her lower extremities' edema improved, and she was only using diuretics as needed.

The patient remained hospitalized for 3 weeks after surgery for postoperative pneumonia and severe deconditioning requiring intensive inpatient rehabilitation. She also developed herpes zoster and severe scalp seborrheic dermatitis 1 month after microadenoma resection.

She was closely monitored as an outpatient after surgery. She has lost 50 pounds of body weight with normalization of potassium level without potassium supplement, and she has had significant improvement in her diabetes control. Her ACTH remained in normal reference range, and her morning cortisol was still in low normal range (Table 3). She still required hydrocortisone 15 mg daily. Her postoperative MRI of the brain, 1 year after surgery, did not show any evidence of recurrence or persistent disease.

**Table 3.** Blood tests, including potassium, hemoglobin A1c, ACTH, and morning cortisol level from the time of initial evaluation, 1 day after surgery, and up to 20 months of follow-up

Tests	Reference range	Initial evaluation	1 day postoperatively	3 months postoperatively	1 year postoperatively	20 months postoperatively
Potassium	3.6-5.2 mEq/L (SI: 3.6-5.2 mmol/L)	2.5 mEq/L (SI: 2.5 mmol/L) (L)	3.4 mEq/L (SI: 3.4 mmol/L) (L)	3.7 mEq/L (SI: 3.7 mmol/L)	4.4 mEq/L (SI: 4.4 mmol/L)	4.1 mEq/L (SI: 4.1 mmol/L)
Hemoglobin A1c	< 5.7%	9.8% (H)	—	—	5.9% (H)	6.0% (H)
ACTH	7.2-63 pg/mL (SI: 1.6-13.9 pmol/L)	264.0 and 309 pg/mL (SI: 58.1 and 68.0 pmol/L) (H)	10 pg/mL (SI: 2.20 pmol/L)	31.8 pg/mL (SI: 7.0 pmol/L)	23.5 pg/mL (SI: 5.17 pmol/L)	22.7 pg/mL (SI: 4.99 pmol/L)
Morning cortisol	7-25 μg/dL (SI: 193-690 nmol/L)	60.4 and 112 μg/dL (SI: 1666 and 3090 nmol/L) (H)	4.6 μg/dL (SI: 126.8 nmol/L) (L)	5.3 μg/dL (SI: 146.23 nmol/L) (L)	4.8 μg/dL (SI: 132.43 nmol/L) (L)	8.7 μg/dL (SI: 240 nmol/L)
24-hour urinary free cortisol (UFC)	3.5-45 μg/24 hours (SI: 9.7-124 nmol/24 hours)	1107 μg/24 hours (SI: 3055 nmol/24 hours) (H)	—	—	—	7.5 μg/24 hours (SI: 21 nmol/24 hours)
Late-night 11 Pm salivary cortisol	< 100 ng/dL (SI: <2.76 nmol/L)	3170 and 4310 ng/dL (SI: 87.48 nmol/L and 118.90 nmol/L) (H)	—	—	—	2.3 ng/dL (SI: 0.64 nmol/L)

Abbreviations: ACTH, adrenocorticotropic hormone; L, low; H, high.

## Discussion

Our case highlights the heterogeneous clinical presentations of CCA with pronounced cortisol hypersecretion that resembles ectopic ACTH syndrome from a pituitary microadenoma. A focal lesion > 6 mm on pituitary MRI in ACTH-dependent Cushing syndrome may provide a definite diagnosis, and no further evaluation may be required [5]. In our case, the size is smaller than the expert consensus, and BIPSS provided a diagnostic value to confirm the central source of ACTH.

The pathophysiology of hypokalemia in Cushing syndrome is thought to be saturation of the enzyme 11-beta-hydroxysteroid dehydrogenase type 2 (11β-HSD2), which converts cortisol to inactive cortisone and prevents the cortisol action on the mineralocorticoid receptors [6, 7].

To this date there is no clear understanding of the pathogenesis for the hyaline change in CCAs. Crooke hyaline change is commonly seen in patients with Cushing syndrome and is correlated with the degree of hypercortisolemia, particularly in patients with high levels of urinary free cortisol (at least 4-fold the upper limit of normal) [8]. The Crooke hyaline change in neoplastic Crooke cells may be secondary to overexpression or lack of downregulation of glucocorticoid receptors. This hypersensitivity to glucocorticoids was previously reported on silent CCAs [9].

Regarding the aggressive clinical behavior for CCAs, high levels of p53 immunoreactivity were found to be related to more frequent recurrences [2]. Single-nucleotide variations in the genes encoding *TP53*, *EGFR*, *HSD3B1,* and *CDKN1A* and low expression of the DNA repair enzyme O^6^-methylguanine-DNA methyltransferase (MGMT) were found to be associated with CCAs as well [10, 11].

Kattah et al reported a case of CCA with similar clinical manifestations. The hormonal evaluation of that case also revealed significantly elevated 24-hour urinary cortisol and morning cortisol more than 30 times above the upper limit of normal, with no suppression with 1 mg dexamethasone. The ACTH level was also significantly elevated. The culprit tumor was found to be a 9- × 6-mm pituitary Crooke cell adenoma. The plasma cortisol, urinary cortisol, ACTH, and potassium levels remained in the normal range up to 2 years of monitoring after surgery [12]. The case reported by Kattah et al was complicated by acute pulmonary thromboembolism in multiple branches requiring therapeutic anticoagulation and was treated with ketoconazole before surgery [12].

Retrospective data revealed increased venous thromboembolism (VTE) risk in patients with active Cushing syndrome, as well as after pituitary and adrenal surgery. The VTE risk could last up to 2 to 3 months or years postoperatively despite biochemical remission [13, 14]. Perioperative prophylaxis for VTE is suggested by society guidelines and could be considered to extend for a longer period after surgery [13, 15].

The mainstay of treatment for CCAs is surgical resection, but the recurrence rate was high. In previous case series, 15 out of 25 patients (60%) were found to have recurrent CCA, and 6 of them (24%) experienced multiple recurrences after 1 year of follow-up, with the time to first recurrence ranging from 1.4 to 8 years (mean 3.6 years) [2]. Patients with functional CCAs frequently required more than one surgery (50%), radiation therapy (59.1%), bilateral adrenalectomy (22.7%), and temozolomide therapy (36.3%) [3]. Medical therapy for Cushing disease may be used in patients with persistent or recurrent disease or patients who are not suitable candidates for surgery. It could also be used in severe disease to rapidly normalize cortisol levels and action [14]. In our case, cortisol-lowering therapy was not given due to the urgency of surgical intervention and rapid deterioration. The patient underwent urgent transsphenoidal tumor resection right after BIPSS results suggested the central source of ACTH.

CCAs were also reported to have less postoperative hormone normalization and remission of hypersecretion despite achieving better local control rates and fewer remnants on MRI compared to other secretory corticotroph adenomas [16].

## Learning Points

- CCAs were more commonly reported as functional invasive macroadenomas with various clinical symptoms. CCA could present as pituitary microadenomas with pronounced cortisol hypersecretion mimicking the features that were more commonly seen in ectopic ACTH syndrome, including hypokalemia and resistant hypertension.
- The pathophysiology underlying the aggressive clinical behavior of CCAs was not clearly understood but may be related to multiple genetic variations that are known to be associated with different tumors and malignancies.
- The mainstay treatment of CCAs is surgical resection. However, CCAs have a higher risk of multiple recurrences even after 1 year of follow-up. Patients frequently need multiple surgeries and additional treatment, including repeat surgery, radiation, and temozolomide.
- CCAs are known to have a poorer prognosis compared to other secretory corticotroph adenomas with less biochemical remission of hypercortisolism and normalization of pituitary function.

## Data Availability

Original data generated and analyzed for this case report are included in this published article.

## Abbreviations

ACTH: adrenocorticotropic hormone
BIPSS: bilateral inferior petrosal sinus sampling
CCA: Crooke cell adenoma
MRI: magnetic resonance imaging
VTE: venous thromboembolism
